# Development of an *ex vivo* xenogeneic bone environment producing human platelet-like cells

**DOI:** 10.1371/journal.pone.0230507

**Published:** 2020-04-07

**Authors:** Shingo Fujiyama, Nobuyasu Hori, Toshiyuki Sato, Shin Enosawa, Mitsuru Murata, Eiji Kobayashi

**Affiliations:** 1 Central Research Laboratories, Sysmex Corporation, Kobe-shi, Hyogo, Japan; 2 Department of Organ Fabrication, Keio University School of Medicine, Tokyo, Japan; 3 Division of Advanced Medical Sciences, National Center for Child Health and Development, Tokyo, Japan; 4 Department of Laboratory Medicine, Keio University School of Medicine, Tokyo, Japan; European Institute of Oncology, ITALY

## Abstract

The efficiency of *in vitro* platelet production is considerably low compared with physiological activity due to the lack of pivotal factors that are essential *in vivo*. We developed an *ex vivo* platelet production system, introducing human megakaryocytes into an isolated porcine thighbone and culturing in closed circuit. The efficiency of the *ex vivo* platelet production system was compared to those *in vivo* and *in vitro*. CD61^+^ platelet-like cells were counted by immunostaining and flow cytometry. Results showed that 4.41 ± 0.27 × 10^3^ CD61^+^ platelet-like cells were produced by 1 × 10^3^ megakaryocytes in the *ex vivo* system, while 3.80 ± 0.87 × 10^3^ and 0.12 ± 0.02 × 10^3^ were produced in the *in vivo* and *in vitro* systems, respectively. Notably, *ex vivo* and *in vitro* production systems generated cells that responded well to thrombin stimulation and expressed functional molecules, such as CD62P. Overall, our *ex vivo* production system was comparable to *in vivo* production system and produced platelet-like cells that were functionally superior to those produced *in vitro*. In future, the present *ex vivo* production system implementing xenogeneic bone marrow would offer a promising alternative for industrial-scale production of platelet-like cells.

## Introduction

Several *in vitro* platelet production systems have been proposed by mimicking *in vivo* environment [1–5]. Physiologically, platelets are hematopoietic-lineage cells. CD34^+^ hematopoietic stem cells differentiate into mature megakaryocytes in the bone marrow niche after multinucleation and cytoplasm enlargement [1, 2]. Subsequently, the mature megakaryocytes migrate in the proximity of bone marrow sinusoids and release proplatelets, which further mature in the sinusoids through the influence of shear stress due to local turbulences, and platelets are secreted into the circulation [3–5]. Along the process, cytokines, scaffold, and intercellular interactions are crucial [6–9]. The number of platelets produced *in vivo* ranges between 1,000 and 5,000 per megakaryocyte; however, the efficiency of *in vitro* production is still low despite considerable efforts and the implementation of thrombopoietin, 3D bioreactors, and artificial turbulence [10, 11].

Thrombopoietin, a cytokine essential for inducing differentiation of hematopoietic stem cells to megakaryocytes, was first cloned in 1994, enabling highly efficient megakaryocyte production [12–16]. Matsunaga *et al*. produced 1.68 × 10^11^ platelets from 5 × 10^6^ CD34^+^ hematopoietic stem cells *in vitro* by optimizing the types and amounts of cytokines added to umbilical cord blood-derived CD34^+^ hematopoietic stem cells [17]. Brent *et al*. produced 14.2 platelets per CD34^+^ hematopoietic stem cell by creating a 3D bioreactor, adding cytokines, and applying shear force [18]. Recently, Ito *et al*. produced 50–100 platelets per megakaryocyte using an immortalized megakaryocytic cell line established from induced pluripotent stem cells and a bioreactor that imitates turbulence generated in bone marrow vessels [19]. These produced platelets were morphologically like platelets *in vivo* and expressed functional molecules in response to induction using ADP.

However, abundant pieces of evidence showed that the platelet production efficiency was low as compared with that *in vivo*. Noteworthy, the incomplete reproduction of cytokines and the scaffolding environment involved in platelet production in the bone marrow niche *in vivo* is a cause of low production efficiency of platelets *in vitro* [19, 20]. Therefore, improvement of production efficiency *in vitro* may be achieved by adding factors effective for platelet production based on analysis of the niche environment *in vivo*.

Herein, we aimed to develop an *ex vivo* production system that has both *in vitro* and *in vivo* characteristics, using isolated porcine bone as a site for production. Moreover, the system will elucidate the detailed mechanisms of platelet differentiation.

## Materials and methods

### Induction of differentiation of human CD34^+^ cells into megakaryocytes

Human Cord Blood CD34^+^ cells, Frozen (StemCell Technologies Inc) were thawed and cultured in StemSpan SEFM II (StemCell Technologies Inc) supplemented with megakaryocyte expansion supplement (StemCell Technologies Inc) and 1% Antibiotic-Antimycotic solution (15240096, Gibco). The medium was exchanged every 3–4 days, and the cell concentration was adjusted to 1–10 × 10^5^ cells/mL.

### Carboxyfluorescein succinimidyl ester (CFSE) labeling of megakaryocytes

Megakaryocytes derived from CD34^+^ cells on the 19th day of culture were washed with phosphate-buffered saline (PBS, FUJIFILM Wako Pure Chemical) and resuspended with PBS to 1 × 10^6^ cells/mL. Then, the megakaryocytes were labeled with 0.1 μg/mL CFSE (CellstainR, C309, Dojindo) for 30 minutes at 37°C. After washing with PBS, the megakaryocytes were resuspended in perfusion medium (RPMI-1640 (R8758, Sigma) supplemented with 10% fetal bovine serum and 1% Antibiotic-Antimycotic solution).

### *In vitro* platelet production system

CFSE-labeled megakaryocytes were incubated at a density of 5 × 10^6^ cells/mL at 37°C for 3 hours, and then platelet-like cells derived from the megakaryocytes in the culture supernatant were collected.

### *In vivo* platelet production system

The protocol for the *in vivo* experiment was conducted with the approval of the Laboratory Animal Ethics Committee of the National Center for Child Health and Development (IRB number: A2000-001) based on the Japanese Guideline for Animal Experiments of Ministry of Health, Labour and Welfare.

**Fig 1:**
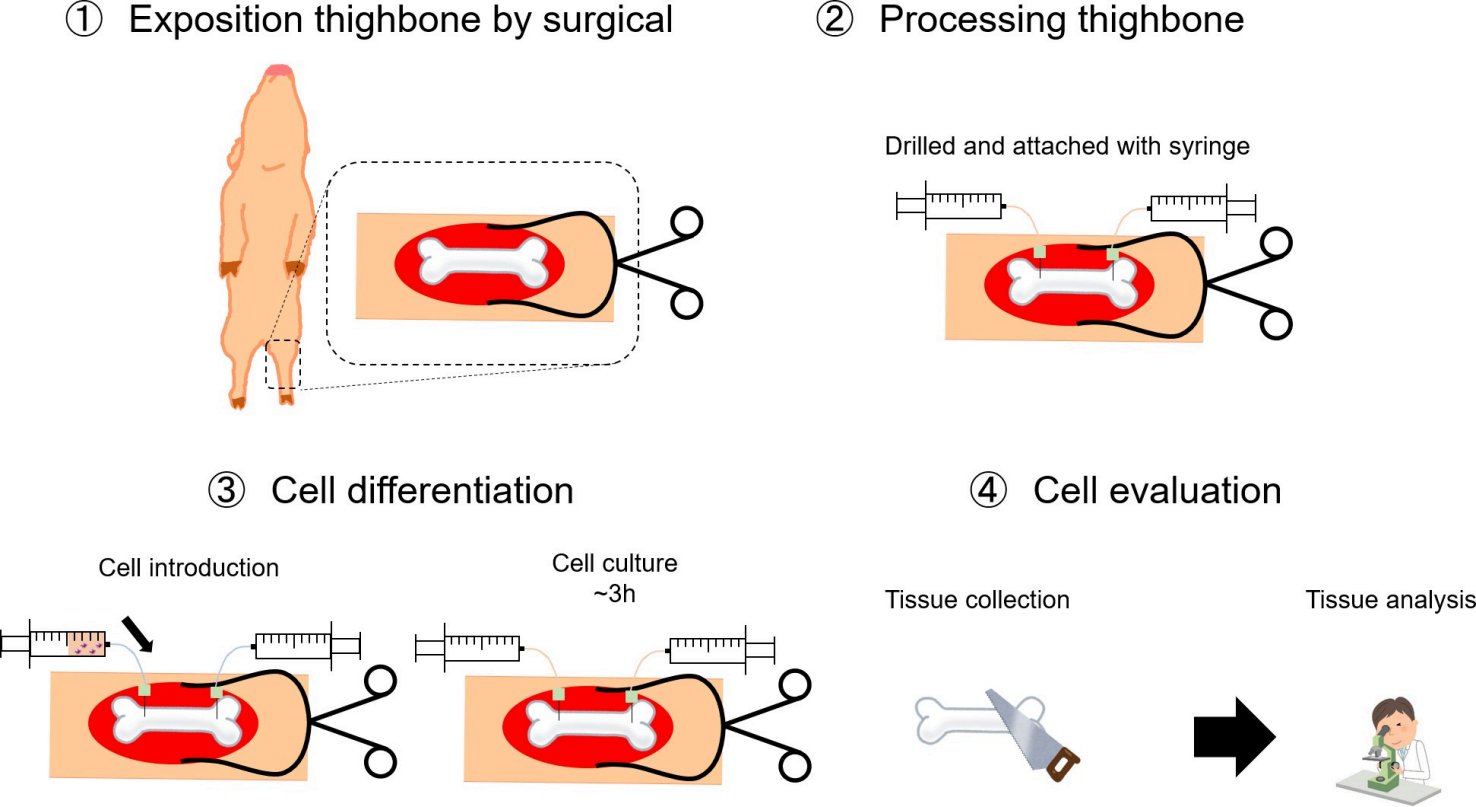
The *in vivo* production system procedure. (1) Porcine thighbone was surgically exposed by using retractors. (2) The thighbone was drilled and attached with syringe. (3) Megakaryocytes were introduced into the thighbone by perfusion and then cultured for 3hours. (4) After harvesting thighbone; bone marrow was frozen and later analyzed by tissue immunohistochemical staining.

We used micro-mini pigs not exceeding 30 kg in weight at the age of 2 years [21]. Twelve-months-old female micro-mini pigs of were purchased from Fuji Micra, Inc., Shizuoka, Japan. Animals were treated per the Animal (Scientific Procedure) Protection Act 1986 of the United Kingdom. The pigs were housed in cages under temperature and light-controlled conditions (12-hour light/dark cycle) and were provided with food and water ad libitum. The pigs fasted for 12 hours before surgery with free access to water. Immunosuppressed pigs were prepared as follows: the micro-mini pigs were intravenously administered mycophenolate mofetil at a dose of 60 mg/kg B.W. daily 5 days before the experiment and tacrolimus at a dose of 0.5 mg/kg B.W. daily 3 days before the experiment. Sedation with a mixture of midazolam/medetomidine/butorphanol was followed by endotracheal intubation and mechanical ventilation. Anesthesia was maintained with inhalational isoflurane. Midazolam and medetomidine were added according to the depth of anesthesia. For the formation of the perfusion pathway to introduce the cells, a hole of 2-mm diameter was drilled at two points in the epiphysis of the thighbone of anesthetized pigs. An 18-G needle attached to a 10-mL syringe with saline was pierced into the hole, and the saline was perfused into the bone marrow with positive pressure while applying negative pressure from another side (Fig 1). Next, 500 μL of CFSE-labeled megakaryocytes (2.5 × 10^6^ cells) were introduced into the thighbone using a 1-mL syringe, followed by 1 mL of saline to remove megakaryocytes in the perfusion line. The introduced megakaryocytes were incubated for 3 hours and subsequently harvested. Buprenorphine was administered as an analgesic for intraoperative pain management. After intravenous administration of pentobarbital saturated potassium chloride was rapidly administered intravenously to euthanize.

### *Ex vivo* platelet production system

**Fig 2:**
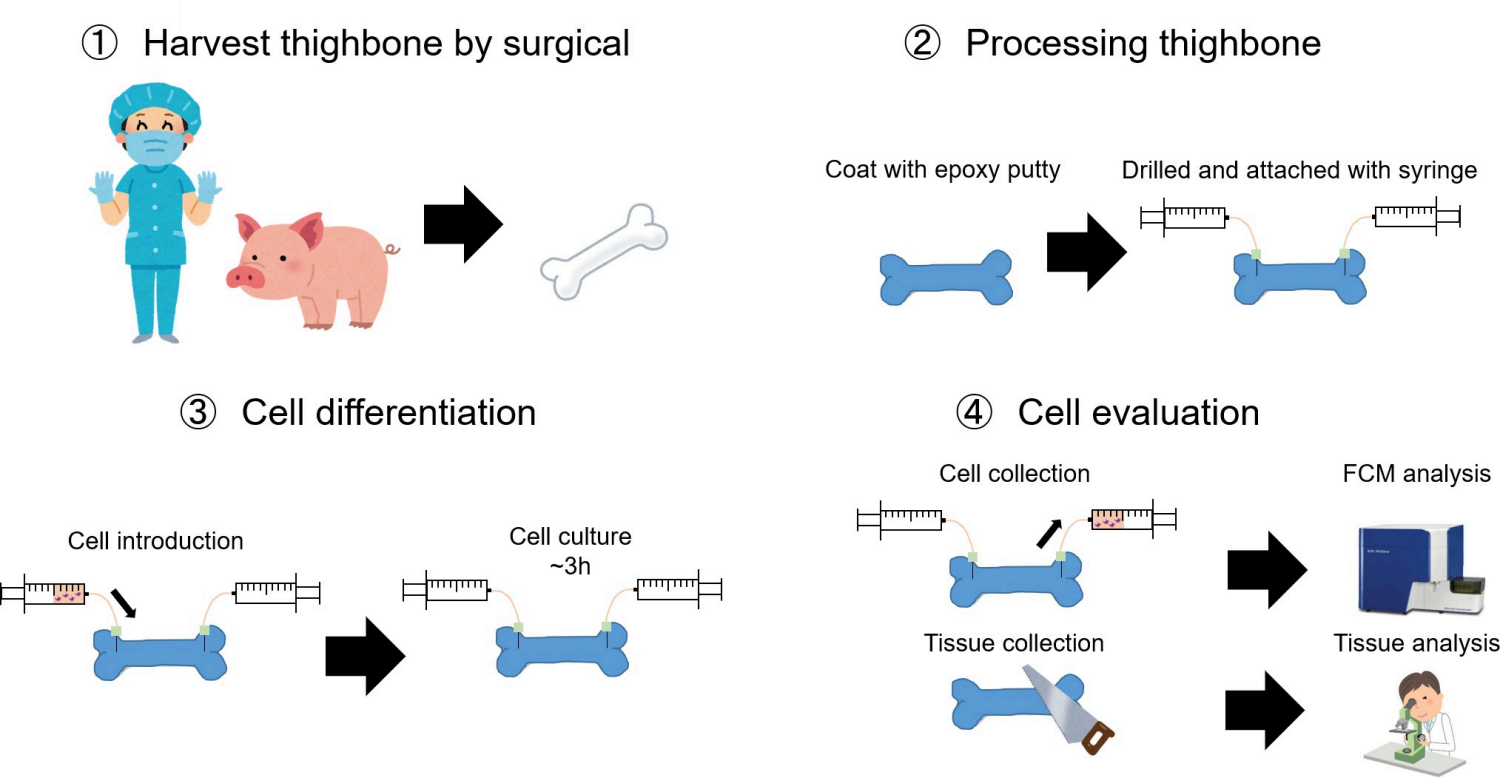
The *ex vivo* production system procedure. (1) Porcine thighbone was surgically harvested. (2) The thighbone was coated with epoxy putty, drilled and attached with syringe. (3) Megakaryocytes were introduced into the thighbone by perfusion and then cultured for 3hours. (4) The produced platelet-like cells were collected by perfusion and then analyzed by flow cytometry. Alternatively, the bone marrow was frozen and later analyzed by tissue immunohistochemical staining.

The protocol for the *ex vivo* experiment was conducted with the approval of the Experimental Animal Ethics Committee of Keio University (IRB number: 14709- (0)) based on Institutional Guidelines on Animal Experimentation at Keio University. The thighbone used for the *ex vivo* production system was harvested from livestock pigs at 3 months old. All animals were also treated in accordance with the Animal (Scientific Procedure) Protection Act 1986 of the United Kingdom as described above in “*In vivo* platelet production system”. The excised thighbone was covered with quick-drying epoxy putty (DHP-482, Loctite) and dried for 20 minutes at room temperature. A hole of 1.3-mm diameter was drilled at two points in the epiphysis of the thighbone. Then, an 18-G needle attached to a 10-mL syringe (syringe B) with perfusion medium was pierced into one hole, and 300 mL of perfusion medium was perfused into the bone marrow with positive pressure at a flow rate of 7 mL/min using a syringe pump while applying negative pressure from the other side (syringe A) using a syringe pump (Fig 2). Next, 500 μL of CFSE-labeled megakaryocytes at a density of 10–40 × 10^6^ cells/mL was introduced into the thighbone and incubated for 3 hours at 37°C for production of platelet-like cells. Following *ex vivo* incubation, 120 mL of perfusion medium was perfused through one hole in the thighbone, and platelet-like cells were collected from the other hole.

### Immunofluorescence of megakaryocytes and immunohistochemistry of platelet-like cells in porcine thighbone

Immunomorphological characterization evaluation of megakaryocytes derived from CD34^+^ cells on the 19th day, cytospin preparation began with 2×10^5^ cells in PBS containing 5% bovin serum albumin and centrifuged at 120 × *g* for 3 minutes and then transferred onto glass slides. Cytospin preparation was fixed by PBS containing 4% paraformaldehyde (PFA) for 10 minutes at room temperature and then incubated with 100 μL of antibody solution (Alexa Fluor 647-labeled anti-CD61 antibody (1:100, BioLegend, clone: VI-PL 2), containing 0.1% Hoechst33342 (Dojindo) in PBS for 10 minutes at room temperature. After washing with PBS, the cytospin preparation was embedded with 50% glycerol in PBS and observed under fluorescence microscope (BZ-X 700, Keyence).

The cancellous bone marrow tissue harvested from the porcine thighbone were embedded in Tissue-Tek OCT compound (Sakura Finetek) and then frozen in dry ice. Frozen tissue sections with a thickness of 10 μm were prepared using a cryostat (Leica Biosystems), immersed in saline for 10 minutes, and then fixed by PBS containing 4% PFA for 10 minutes at room temperature. After washing with saline, the sections were incubated with 100 μL of antibody solution (Alexa Fluor 647-labeled anti-CD61 antibody (1:100), containing 0.1% Hoechst33342 in PBS for 10 minutes at room temperature. After washing with PBS, the sections were embedded with 50% glycerol in PBS and observed under fluorescence microscope, and the number of CD61^+^ platelet-like cells was counted.

### Flow cytometric analysis of platelet-like cells

This study was approved by Sysmex Ethics Committee. All participants provided their written informed consent to participate in this study according to the study protocol. To define a platelet FSC-SSC scattergram, platelets from healthy volunteer donors were first evaluated by flow cytometry. Blood samples were placed into blood collection tubes containing acid-citrate-dextrose (Becton, Dickinson and Company) and then centrifuged at 200 × *g* for 10 minutes to prepare platelet-rich plasma (PRP). Then; 5 μL of PRP was added to 95 μL of PBS and evaluated using FACSVerse (BD Biosciences).

Cells were centrifuged at 200 × *g* for 10 minutes and washed with PBS. Then the cells were fixed with 1% PFA in PBS for 10 minutes and centrifuged at 200 × *g* for 10 minutes. After washed with PBS, 5 μL of antibody (allophycocyanin-labeled anti-CD42b antibody (BioLegend, clone: ​​HIP-1), PerCP-Cy5.5 -labeled anti-CD61 antibody (BD Biosciences, clone: VI-PL2), allophycocyanin-labeled mouse IgG1 antibody (R&D Systems, clone: 11711), or PerCP-Cy5.5-labeled mouse IgG1 antibody (BD Biosciences, clone: ​X-40) was added to 100 μL of cell suspension respectively and incubating for 15 minutes at room temperature. After the reaction, the cells were washed with PBS and were evaluated using FACSVerse.

For platelet marker analysis, the cells harvested from *ex vivo*, *in vitro*, and *in vivo* production systems were fixed with 1% PFA in PBS for overnight and centrifuged at 200 × *g* for 10 minutes. After the supernatant was collected, the supernatant was centrifuged at 1500 × *g* for 10 minutes and washed with PBS. Then 5 μL of antibody (allophycocyanin-labeled anti-CD42b antibody, Alexa Fluor 647-labeled anti-CD61 antibody, allophycocyanin-labeled mouse IgG1 antibody, or Alexa Fluor 647-labeled mouse IgG1 antibody (BioLegend, clone: ​MOPC-21)) was added to 100 μL of cell suspension that was incubated for 15 minutes at room temperature. After the reaction, the cells were washed with PBS, and evaluated using FACSVerse. The number of platelet-like cells was calculated using counting beads as external standard (C36950, Thermo Fisher SCIENTIFIC).

For functional molecule expression analysis, cells harvested from *ex vivo* and *in vitro* production systems were centrifuged at 200 × *g* for 10 minutes. The supernatant was collected, mixed with 0.5 μM prostaglandin I2 (P6188, Sigma-Aldrich), and centrifuged at 1500 × *g* for 10 min. The supernatant was removed, and the cells were suspended in Tyrode's buffer (134 mM NaCl, 0.34 mM Na_2_HPO_4_, 2.9 mM KCl, 12 mM NaHCO_3_, 20 mM HEPES, 5 mM glucose, and 1 mM MgCl_2_). Next, 5 μL of antibody (allophycocyanin-labeled anti-CD62P antibody (BioLegend, clone: AK-4) or allophycocyanin-labeled mouse IgG1 antibody) was added to 100 μL of cell suspension, respectively. To test samples, 1 U/mL thrombin (T9326, Sigma-Aldrich) and 2.5 mM Ca^2+^ were added. The cells were incubated for 15 minutes at room temperature, fixed with 1% paraformaldehyde in PBS overnight, and analyzed by FACSVerse.

## Results

CD34^+^ cells were differentiated into megakaryocytes and measured megakaryocyte marker over time. CD42b^+^CD61^+^ cells were detectable at day 7 and increased to 56.7% at day 19 (S1A, S1C and S1D Fig). In addition, on day 19, the megakaryocytes had undergone polyploidization, and some were proplatelets (S1B Fig). Thus, we used the CFSE-labeled cells at day 19 as megakaryocytes to evaluate the platelet differentiation by *in vitro*, *in vivo*, and *ex vivo* production system.

**Fig 3:**
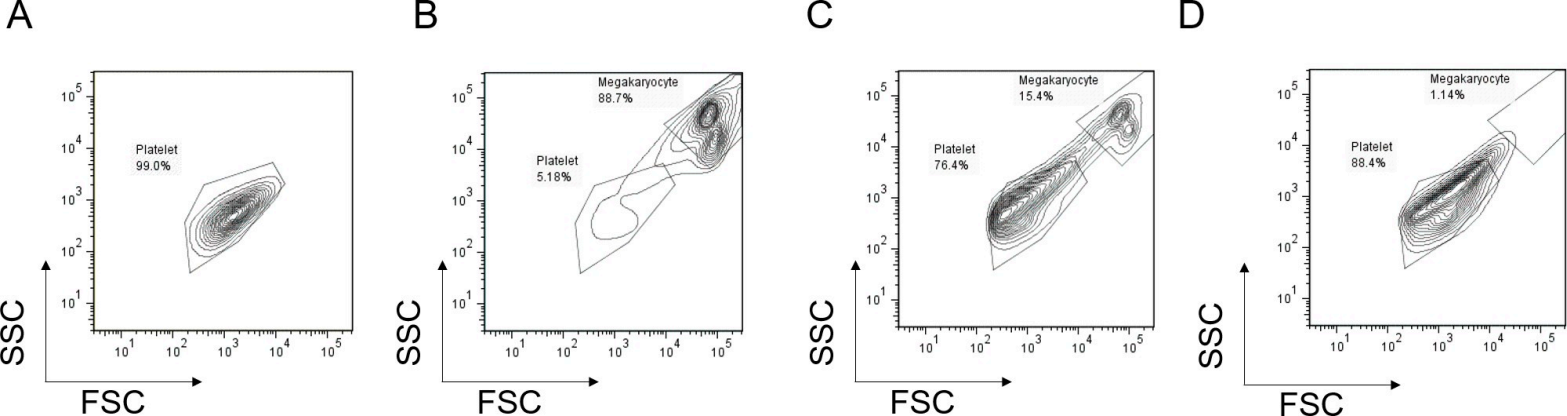
Flowcytometric profile of cells. (A) Platelets of healthy volunteer donor. (B) Megakaryocytes prior to platelet-like cells production. (C) Platelet-like cells produced by *in vitro* production system. (D) Platelet-like cells produced by *ex vivo* production system.

In all experiments, platelet-like cells derived from CFSE-labeled megakaryocytes were defined as cells that are same size as platelets and positive for CFSE (Fig 3A). Initially, cells collected by *in vitro* production system were assessed by flow cytometry. *In vitro*, the rate of platelet-like cells among all collected CFSE^+^ cells was 66.3 ± 11.9% (Table 1, Fig 3B and 3C). In addition, the rates of CD61^+^ and CD42b^+^ platelet-like cells in all platelet-like cells were 79.7% and 5.8%, respectively. As for platelet function, the rate of CD62P^+^ platelet-like cells with or without thrombin stimulation was determined. CD62P^+^ platelet-like cells were increased from 32.2% under no stimulation to 45.3% under thrombin stimulation (Table 1). Furthermore, the production number of CD61^+^ platelet-like cells was 124 ± 22 per 1 × 10^3^ megakaryocytes (Table 1).

**Table 1:** The rate of CD42b^+^ and CD61^+^ cells and the number of platelet-like cells produced by each production system.

	Production systems		
	*In vitro*	*In vivo*	*Ex vivo*
Percentage of platelet-like cells by flow cytometry ^a^			
CFSE^+^ cells of all cells (%)	79.3 ± 25.5	n.d. ^b^	0.85 ± 0.73
FSC-SSC plot of CFSE^+^ cells (%)	66.3 ± 11.9	n.d.	63.0 ± 19.9
CD42b^+^ cells of FSC-SSC plot (%)	5.8 ± 1.7	n.d.	14.0 ± 4.5
CD61^+^ cells of FSC-SSC plot (%)	79.8 ± 3.0	n.d.	76.9 ± 0.9
Responsiveness of platelet-like cells to thrombin stimulation ^c^			
CD62P^+^ cells without thrombin (%)	32.2	n.t. ^d^	53.8
CD62P^+^ cells with thrombin (%)	45.3	n.t.	62.5
Increase (%)	13.1	n.t.	8.7
Number of platelet-like cells ^a^			
By flow cytometry (CFSE^+^ and FSC-SSC plot)	160 ± 28	n.d.	92 ± 49
By flow cytometry (CD61^+^ staining)	124 ± 22	n.d.	65 ± 34
By microscopy (CD61^+^ staining)	n.t.	3,795 ± 872 ^e^	4,411 ± 271

a. Data are expressed as mean ± SD, n = 3, except for “e.”

b. n.d.; not detected

c. by flow cytometry

d. n.t.; not tested

e. mean ± range, n = 2

**Fig 4:**
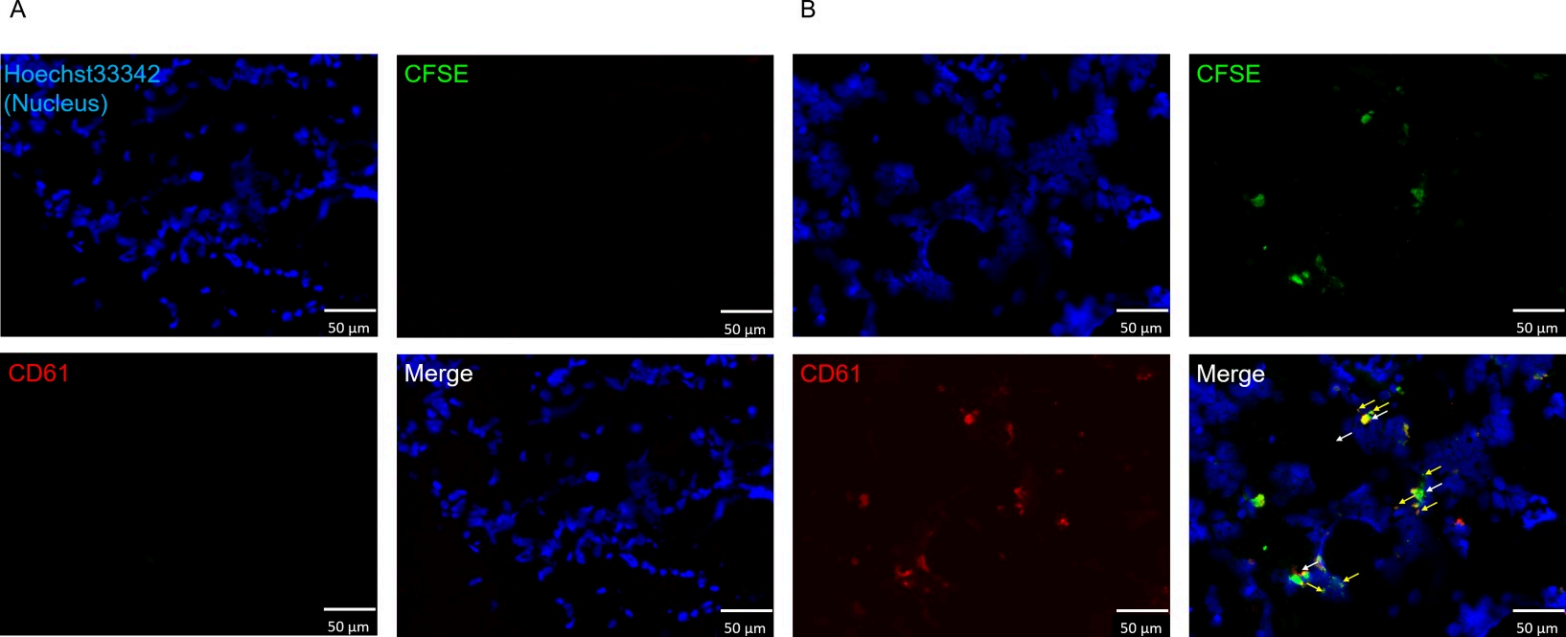
**Immunohistochemical staining of thighbone using *in vivo* production system (A) with or (B) without megakaryocytes introduction.** The properties of platelet-like cells produced by the *in vivo* production system were evaluated by immunohistochemical staining of the porcine thighbone. White arrows indicate introduced megakaryocytes while yellow arrows indicate CFSE-labeled, CD61+ platelet-like cells. Hoechst33342, CFSE, and anti-CD61 antibody are shown in blue, green, and red, respectively.

In *in vivo*, the production number of platelet-like cells was evaluated by immunohistochemical staining of the harvested thighbone. CD61^+^ platelet-like cells were observed around introduced megakaryocytes (Fig 4). Two porcine were used for the experiment, and the number of CD61^+^ platelet-like cells produced from the 1 × 10^3^ megakaryocytes introduced for each was 4666 and 2923 (Table 1). When sections of bone marrow at both ends and center were observed, megakaryocytes and platelets were present at all sites (S3 Fig). Therefore, this suggest that the introduced megakaryocytes are distributed throughout the bone marrow. Besides, platelet-like cells were not detected from peripheral blood.

**Fig 5:**
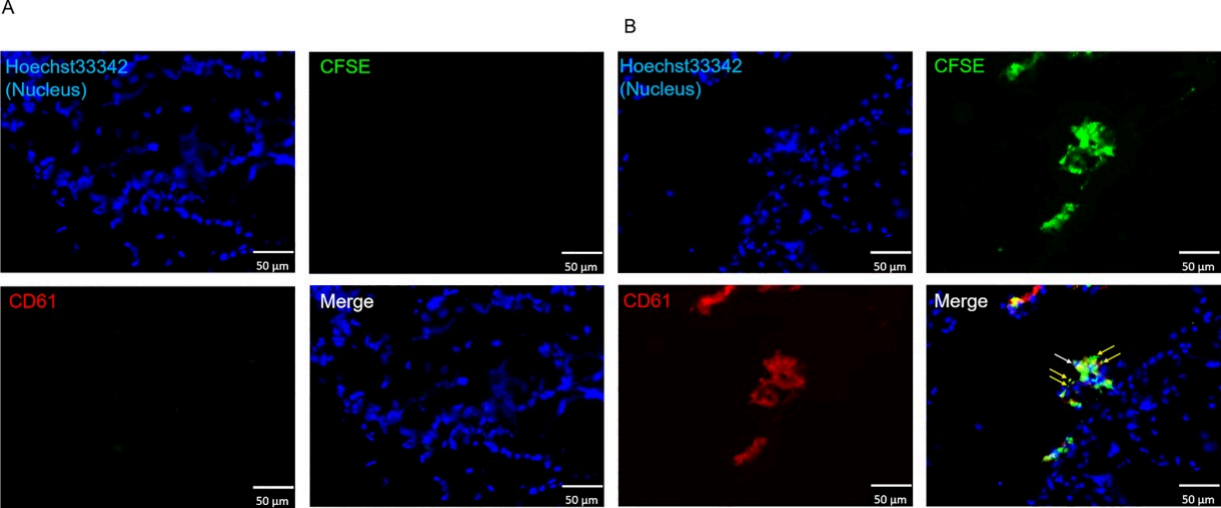
**Immunohistochemical staining of thighbone using *ex vivo* production system (A) with or (B) without megakaryocytes introduction.** The properties of platelet-like cells produced by the *ex vivo* production system were evaluated by immunohistochemical staining of the porcine thighbone. White arrows indicate introduced megakaryocytes while yellow arrows indicate CFSE-labeled, CD61+ platelet-like cells. Hoechst33342, CFSE, and anti-CD61 antibody are shown in blue, green, and red, respectively.

We developed an *ex vivo* platelet production system utilizing a natural biological environment, porcine thighbone, in which a perfusion line was formed to introduce and collect cells. To verify the production efficiency of platelet-like cells from megakaryocytes in the *ex vivo*, the properties and number of produced platelet-like cells were evaluated and compared with those of the *in vitro* and *in vivo*. Initially, cells collected by the *ex vivo* production system were assessed by flow cytometry. Most of the collected cells were pig-derived blood cells, and the introduced megakaryocyte-derived CFSE^+^ cells were 0.85% ± 0.73% (Table 1 and S2 Fig). The rate of platelet-like cells in all collected CFSE^+^ cells was 63.0 ± 19.9% (Table 1 and Fig 3D). In addition, the rates of CD61^+^ and CD42b^+^ platelet-like cells in all platelet-like cells were 76.9% and 14.0%, respectively (Table 1). The number of CD61^+^ platelet-like cells produced from 1 × 10^3^ megakaryocytes was 65 ± 34, and the rate of CD62P^+^ platelet-like cells in all of platelet-like cells increased from 53.8% to 62.5% by thrombin stimulation (Table 1). Therefore, the platelet-like cells produced by the *ex vivo* production system responded to the stimulation. Next, platelets in the bone marrow were evaluated. The number of platelet-like cells was evaluated by immunohistochemical staining of bone marrow after *ex vivo* production. Similar to the results of the ex vivo production system, CD61^+^ platelet-like cells were observed around introduced megakaryocytes, and the number of CD61^+^ platelet-like cells produced from 1 × 10^3^ megakaryocytes was 4411 ± 271 (Table 1, Fig 5). When the bone marrow sections at both ends and center were observed, megakaryocytes and platelets were present at all sites (S3 Fig). Therefore, this suggest that the introduced megakaryocytes are distributed throughout the bone marrow.

These results showed that platelet-like cells produced by the *ex vivo* production system had a CD61^+^ rate equal to that obtained by the *in vitro* production system and a higher CD42b^+^ rate than that achieved with the *in vitro* production system. Furthermore, the number of platelet-like cells produced by the *ex vivo* production system was higher than that produced by the *in vitro* production system and equal to that produced by the *in vivo* production system (Table 1). These results suggested that the *ex vivo* production system more efficiently produces CD61^+^ and CD42b^+^ platelet-like cells than the *in vitro* production system, and it is possible to collect and analyze platelet-like cells produced with the same efficiency as the *in vivo* production system.

## Discussion

In this study, we compared the efficiency of *in vitro*, *in vivo* and *ex vivo* platelet production systems. The number of platelet-like cells produced *in vitro* were counted by flow cytometry, while the platelet-like cells produced *in vivo* and *ex vivo* were counted under a microscope. It is not common to count the number of produced platelet-like cells using microscopic images. When platelets of volunteer blood were introduced and collected in the *ex vivo* production system, the collection efficiency was 0.91%. On counting by the flow cytometry method and the recovery efficiency, the number of platelet-like cells produced in the thighbone was 7.10 × 10^3^ per 1 × 10^3^ megakaryocytes. This number is similar to the number counted by microscopy. Therefore, the method of counting the number of platelet-like cells produced using a microscope image is considered to be valid. The number of CD61^+^ platelet-like cells produced per 1 × 10^3^ megakaryocytes by the *in vitro*, *in vivo*, and *ex vivo* production systems was 124, 3795, and 4411, respectively.

Thus, the *in vivo* and *ex vivo* and production systems can produce platelet-like cells with higher efficiency than the *in vitro* production system. This result suggests that factors that promote production from megakaryocytes to platelets exist in the bone marrow environment used in the *in vivo* and *ex vivo* production systems. In addition, in the *ex vivo* production system, cells are quiesced after cell introduction to induce differentiation, and thus, it is considered that the cells are not subjected to mechanical stimulation such as turbulence. Therefore, we believe that biological factors in the porcine bone marrow environment promoted platelet production. The *ex vivo* production system produced platelet-like cells with a higher CD42b^+^ rate than the *in vitro* production system and at a number comparable to that of the *in vivo* production system. In the *in vitro* production system, CD42b is known to be cleaved by ADAM-17 when the production of megakaryocytes to platelets is carried out at 37°C. Therefore, it is challenging to obtain platelets highly expressing CD42b [22]. On the other hand, because the *ex vivo* production system produced platelet-like cells with a high CD42b^+^ rate, there may have been factors inhibiting the cleavage of CD42b, including by ADAM-17, in the bone marrow environment or factors that enhanced the expression of CD42b.

Matsunaga *et al*. reported that a single megakaryocyte differentiated from umbilical cord blood-derived CD34^+^ cells produced 4 platelets in 5 days by using *in vitro* production system [17]. Nakamura *et al*. reported that a single megakaryocyte differentiated from iPS cells produced 3–10 platelets in 5 days from megakaryocytes induced to differentiate by using *in vitro* production system [23]. Tozawa *et al*. reported that a single megakaryocyte differentiated from adipose-derived mesenchymal stem/stromal cells produced 5–10 platelets in 12 days by using *in vitro* production system [24]. These are calculated by the production efficiency per 3 hours, and it is shown that they produce 100, 70–250, and 52–104 platelets per 1 × 10^3^ megakaryocytes, respectively. In other words, the platelet production efficiency was comparable to that of the *in vitro* production system. Moreover, Matsubara *et al*. reported that a single megakaryocyte differentiated from adipose-derived mesenchymal stem/stromal cells transplanted to mice produced 5–10 platelets 3 hours after transplantation [25]. The production efficiency was suggested to be about the same as *in vivo* production system in this study also produces 2–5 platelets per megakaryocyte in 3 hours. Therefore, *ex vivo* production system has almost the same platelet production efficiency as existing *in vivo* production system and can produce more platelets at the same time, as compared with existing *in vitro* production systems. It is known that platelets collected from a living body deteriorate in about 4 to 5 days and fail to meet blood transfusion criteria. Therefore, when platelets are produced *ex vivo*, a system for producing them in copious amounts in a brief time is required. The platelet production efficiency per 3 hours of the *ex vivo* production system we developed is 35 times that of the *in vitro* production system, so it is useful as a system for producing functional platelets. However, the collection efficiency of the produced platelet-like cells was low with the *ex vivo* production system. By comparing the number of platelets collected from the thighbone by perfusion with that introduced into thighbone, the collection efficiency of platelets by perfusion was found to be only 0.91%. Furthermore, CFSE^+^ platelet-like cells rate of all collected cells from *ex vivo* production system was 0.44 ± 0.32%. Therefore, even if the collection efficiency is 100%, only 33% of the cells are human platelet-like cells, and most of them are cells derived from pig. To collect only human platelet-like cells, a technique for separating pig cells and human platelet-like cells is required. Therefore, the *ex vivo* production system is expected to improve platelet collection efficiency and to develop cell separation techniques. Alternatively, efficient platelet production could be achieved by elucidating two factors that may play a vital role in the mass production of platelet-like cells and inhibiting the cleavage of CD42b in the *ex vivo* production system and adding them to the *in vitro* production system. In the *ex vivo* production system, it is possible to verify which elements are essential for the production by selectively removing them from the tissue or adding of factors using tissue engineering techniques, such as decellularization and perfusion culture [26]. Therefore, unlike the *in vitro* production system, in which platelet production factors are supplemented to cells, we believed that the *ex vivo* production system is useful for screening factors that influence platelet production, as it is possible to exclude factors that are verified not to be related to production [27].

In conclusion, we verified the usefulness of the *ex vivo* production system for mass production of platelet-like cells from megakaryocytes. We clarified that there are some issues in *ex vivo* production system that need to be resolved before realizing industrial-scale production of platelet-like cells. Besides, identification of critical production factors using this system will enable the improvement of current *in vitro* production systems.

## Supporting information

S1 Fig CD34+ cells differentiated into megakaryocytes on day 19 of culturing. (A) Time-course changes of CD61^+^ and CD42b^+^ cell ratios when CD34+ cells were induced to differentiate into megakaryocytes (n = 3, average ± SD). (B) (a) Immunohistochemical staining of the megakaryocytes. Hoechst33342 and anti-CD61 antibody staining are shown in blue and red, respectively. (b) Bright-field images of the megakaryocytes. Black arrows indicate proplatelets. (C) Representative flow cytometry plots of surface molecule expression on cells differentiated from CD34+ cells on day 19 (a) isotypic control antibody (b) anti-cell surface marker antibody. The y-axes indicate CD61, while the x-axes indicate CD42b expression. The left panel shows isotype control, and the right panel shows the antibody. (D) Non-labeled (red line) and CFSE-labeled (blue line) megakaryocytes were analyzed using a flow cytometer. The y-axes indicate count rate; the x-axes indicate CFSE intensity.
(TIF)

S2 Fig Analysis of properties of collected cells of the *ex vivo* production system. Upper row, megakaryocytes were not administered into the thighbone; lower row, megakaryocytes were administered into the thighbone. (a, c) FSC-SSC plot of all collected cells. (b, d) FSC-CFSE-Fluorescence plot of all collected cells. (c, e) FSC-SSC plot of CFSE^+^ cells.
(TIF)

S3 Fig **Immunohistochemical staining of thighbone using *in vivo* or *ex vivo* production system (A) with or (B) without megakaryocytes introduction.** The properties of platelet-like cells produced by the *in vivo* production system were evaluated by immunohistochemical staining of the porcine thighbone. White arrows indicate introduced megakaryocytes while yellow arrows indicate CFSE-labeled, CD61+ platelet-like cells. Hoechst33342, CFSE, and anti-CD61 antibody are shown in blue, green, and red, respectively.
(TIF)
