# Supplementary figures and images for: Development of an ex vivo xenogeneic bone environment producing human platelet-like cells
Source: PLoS One. 2020 Apr 7;15(4):e0230507. doi: 10.1371/journal.pone.0230507 (PMC7138292; doi:10.1371/journal.pone.0230507)

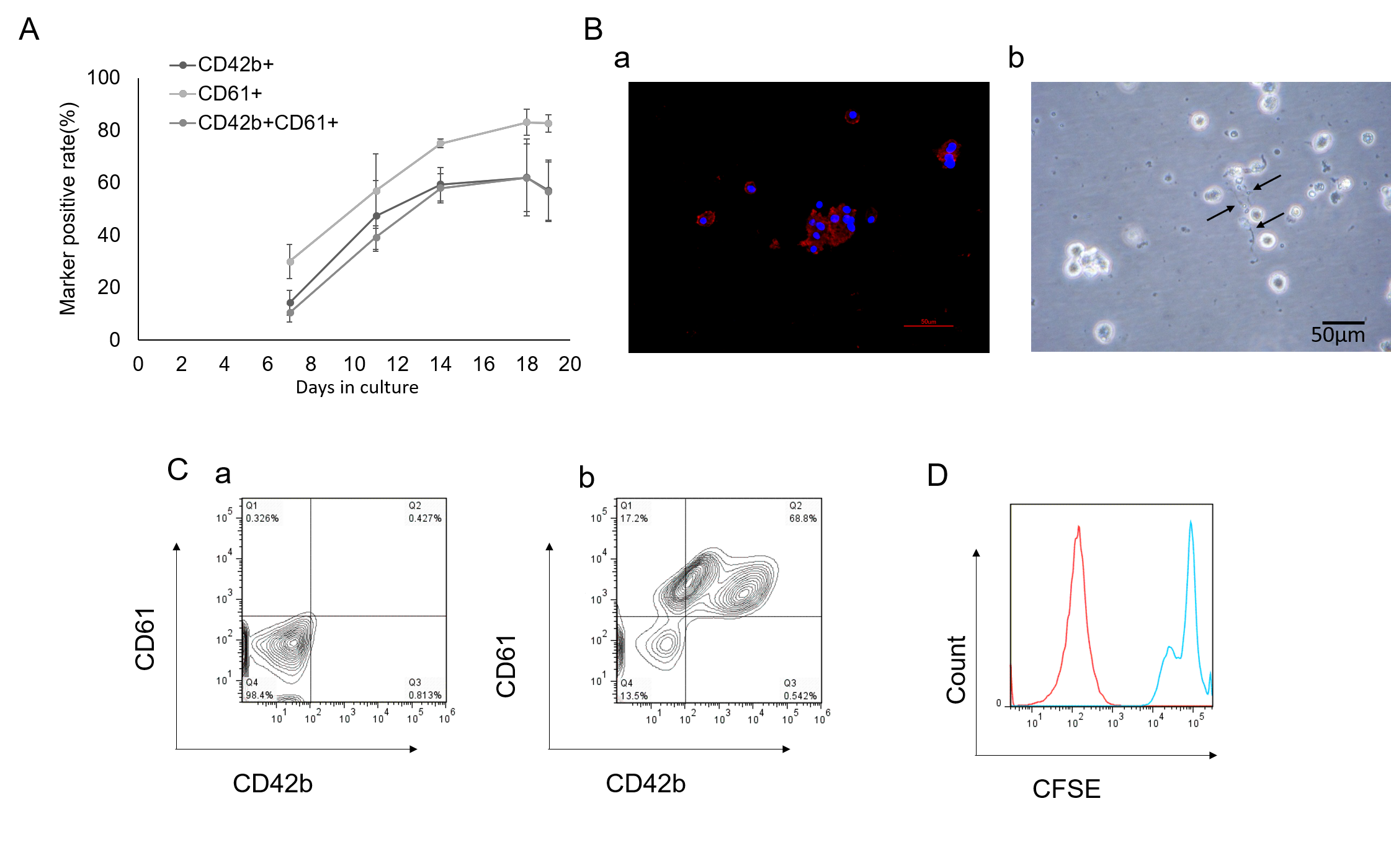

Supplement: S1 Fig — (A) Time-course changes of CD61+ and CD42b+ cell ratios when CD34+ cells were induced to differentiate into megakaryocytes (n = 3, average ± SD). (B) (a) Immunohistochemical staining of the megakaryocytes. Hoechst33342 and anti-CD61 antibody staining are shown in blue and red, respectively. (b) Bright-field images of the megakaryocytes. Black arrows indicate proplatelets. (C) Representative flow cytometry plots of surface molecule expression on cells differentiated from CD34+ cells on day 19 (a) isotypic control antibody (b) anti-cell surface marker antibody. The y-axes indicate CD61, while the x-axes indicate CD42b expression. The left panel shows isotype control, and the right panel shows the antibody. (D) Non-labeled (red line) and CFSE-labeled (blue line) megakaryocytes were analyzed using a flow cytometer. The y-axes indicate count rate; the x-axes indicate CFSE intensity. (TIF) [file pone.0230507.s001.tif]

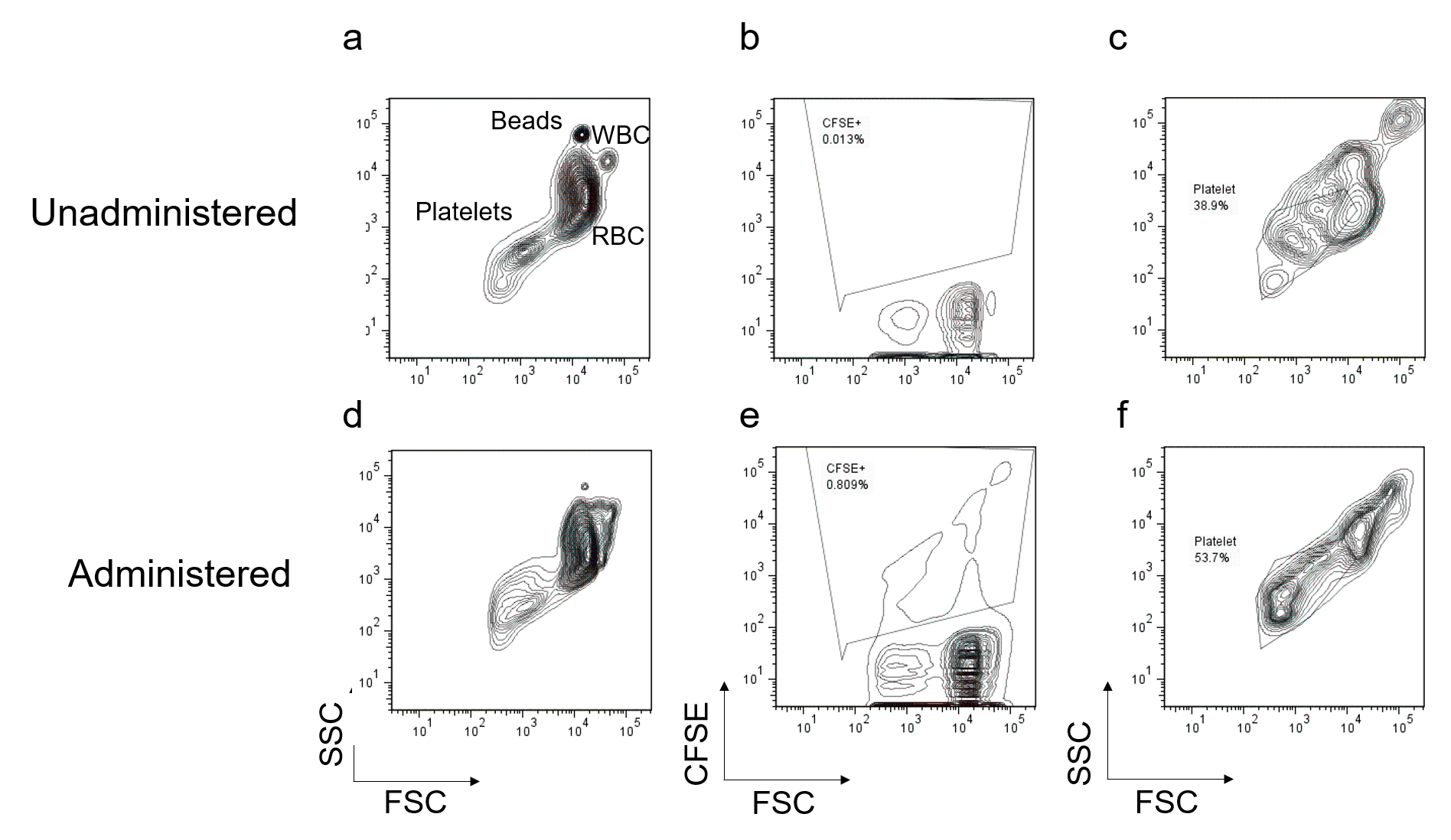

Supplement: S2 Fig — Upper row, megakaryocytes were not administered into the thighbone; lower row, megakaryocytes were administered into the thighbone. (a, c) FSC-SSC plot of all collected cells. (b, d) FSC-CFSE-Fluorescence plot of all collected cells. (c, e) FSC-SSC plot of CFSE+ cells. (TIF) [file pone.0230507.s002.tif]

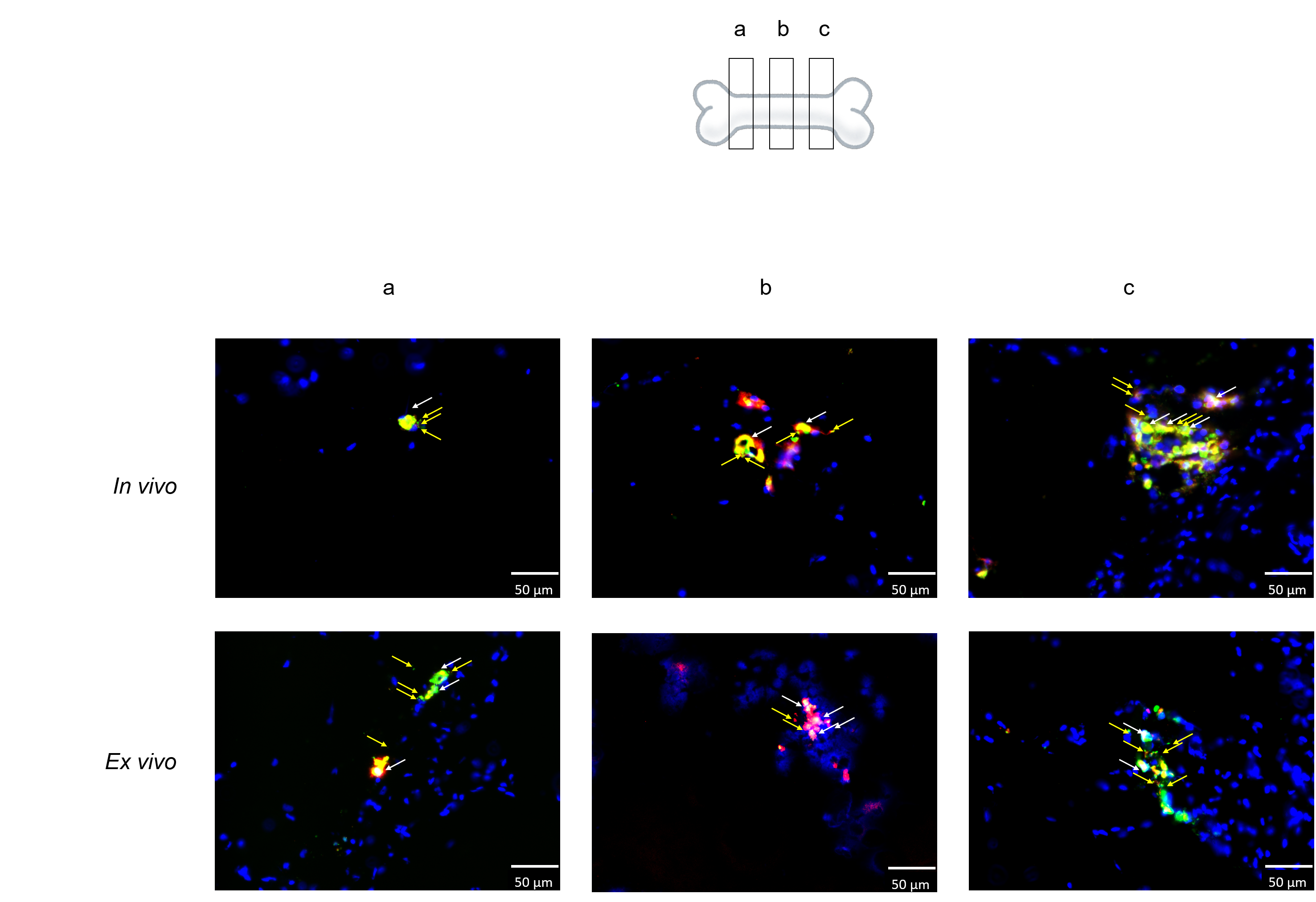

Supplement: S3 Fig — Immunohistochemical staining of thighbone using in vivo or ex vivo production system (A) with or (B) without megakaryocytes introduction. The properties of platelet-like cells produced by the in vivo production system were evaluated by immunohistochemical staining of the porcine thighbone. White arrows indicate introduced megakaryocytes while yellow arrows indicate CFSE-labeled, CD61+ platelet-like cells. Hoechst33342, CFSE, and anti-CD61 antibody are shown in blue, green, and red, respectively. (TIF) [file pone.0230507.s003.tif]
